# Supplementary material for: Systemic Therapy in Advanced Nodular Melanoma versus Superficial Spreading Melanoma: A Nation-Wide Study of the Dutch Melanoma Treatment Registry
Source: Cancers (Basel). 2022 Nov 19;14(22):5694. doi: 10.3390/cancers14225694 (PMC9688066; doi:10.3390/cancers14225694)
Supplement: Supplementary file 1 [file cancers-14-05694-s001.zip › cancers-2007889-supplementary.pdf]

Table S1. Multi variable treatment related cox regression distant metastasis free survival analysis.

Variables		N	Hazard ratio - 95% CI	P-value
Age		3302	0.83 (0.77 – 0.90)	**<0.01**
Gender	Male	2030 (61)	Reference	
	Female	1302 (39)	0.83 (0.74 - 1.06)	0.19
WHO	0 - 1	2792 (84)	Reference	
	2 - 4	59 (11)	1.02 (0.99 - 1.06)	0.20
Melanoma type	SSM	2246 (67)	Reference	
	NM	1086 (33)	1.32 (1.20 – 1.45)	**<0.01**
Ulceration	Absent	1825 (55)	Reference	
	Present	1191 (36)	1.12 (1.09 – 1.15)	**0.04**
Breslow Thickness	0.1 – 1.0mm	457 (14)	Reference	
	1.0 – 2.0 mm	948 (29)	0.91 (0.79 – 1.04)	0.18
	2.0 – 4.0mm	1064 (32)	1.01 (0.91 – 1.15)	0.07
	>4.0mm	863 (26)	1.25 (1.18 – 34)	**0.01**
Satellite lesions	Absent	2635 (79)	Reference	
	Present	334 (10)	0.98 (0.97 – 1.01)	0.18
Dermal mitosis	Absent	307 (9)	Reference	
	Present	1850 (56)	0.94 (0.93 – 0.95)	**<0.01**
BRAF mutation	Absent	1058 (33)	Reference	
	Present	2116 (66)	0.96 (0.89 – 1.02)	0.19
NRAS mutation	Absent	1916 (77)	Reference	
	Present	582 (23)	0.98 (0.91 – 1.05)	0.55

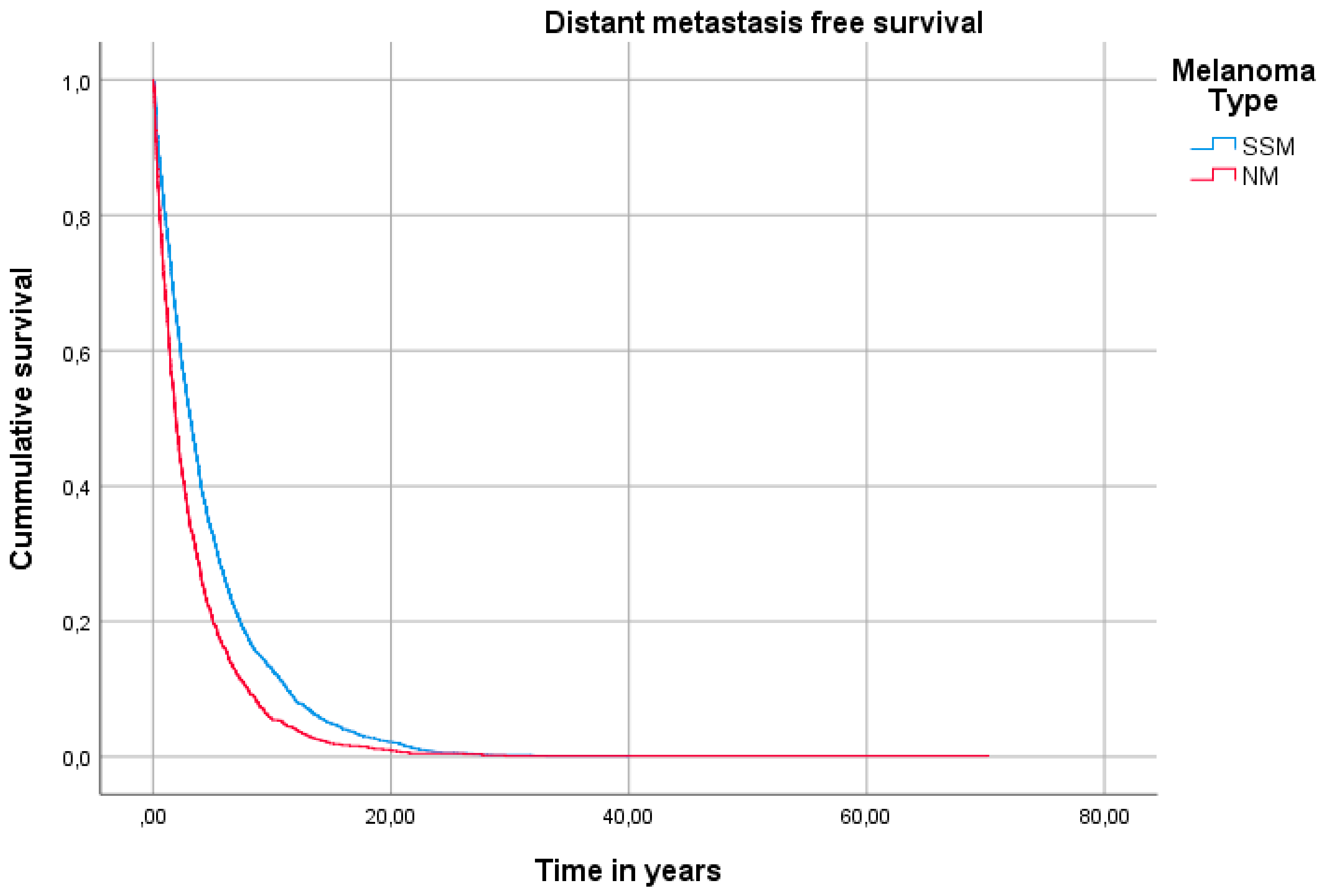

Figure S1. Kaplan Meier curve distant metastasis free survival analysis demonstrating the cumulative survival of NM (red) versus SSM (blue).

Table S2. Multi variable treatment related cox regression progression free survival analysis in patients treated with immune checkpoint inhibition.

Variables		N	Hazard ratio - 95% CI	P-value
Age		2104	1.00 (0.99 – 1.00)	0.91
Gender	Male	1333	Reference	
	Female	771	0.83 (0.70 – 0.97)	**0.02**
WHO	0 - 1	1902	Reference	
	2 - 4	79	1.02 (0.98 – 1.05)	0.32
Treatment type	Anti-CTLA-4	462	Reference	
	Ant-PD-1 / Anti-CLTA-4	1642	0.91 (0.81 – 1.02)	0.10
LDH	Not elevated	1609	Reference	
	Elevated	458	1.22 (1.13 – 1.32)	**<0.01**
Cerebral disease	Absent	1713	Reference	
	Present	351	1.05 (1.01 - 1.11)	**0.03**
Total organ sites	<3	678	Reference	
	>2	1137	1.01 (0.87 - 1.20)	0.82
Melanoma	SSM	1357	Reference	
	NM	747	0.87 (0.74 – 1.02)	0.09
BRAF mutation	Absent	1053	Reference	
	Present	916	0.85 (0.74 – 0.97)	**0.02**
NRAS mutation	Absent	1112	Reference	
	Present	574	1.12 (1.01 – 1.24)	**0.03**

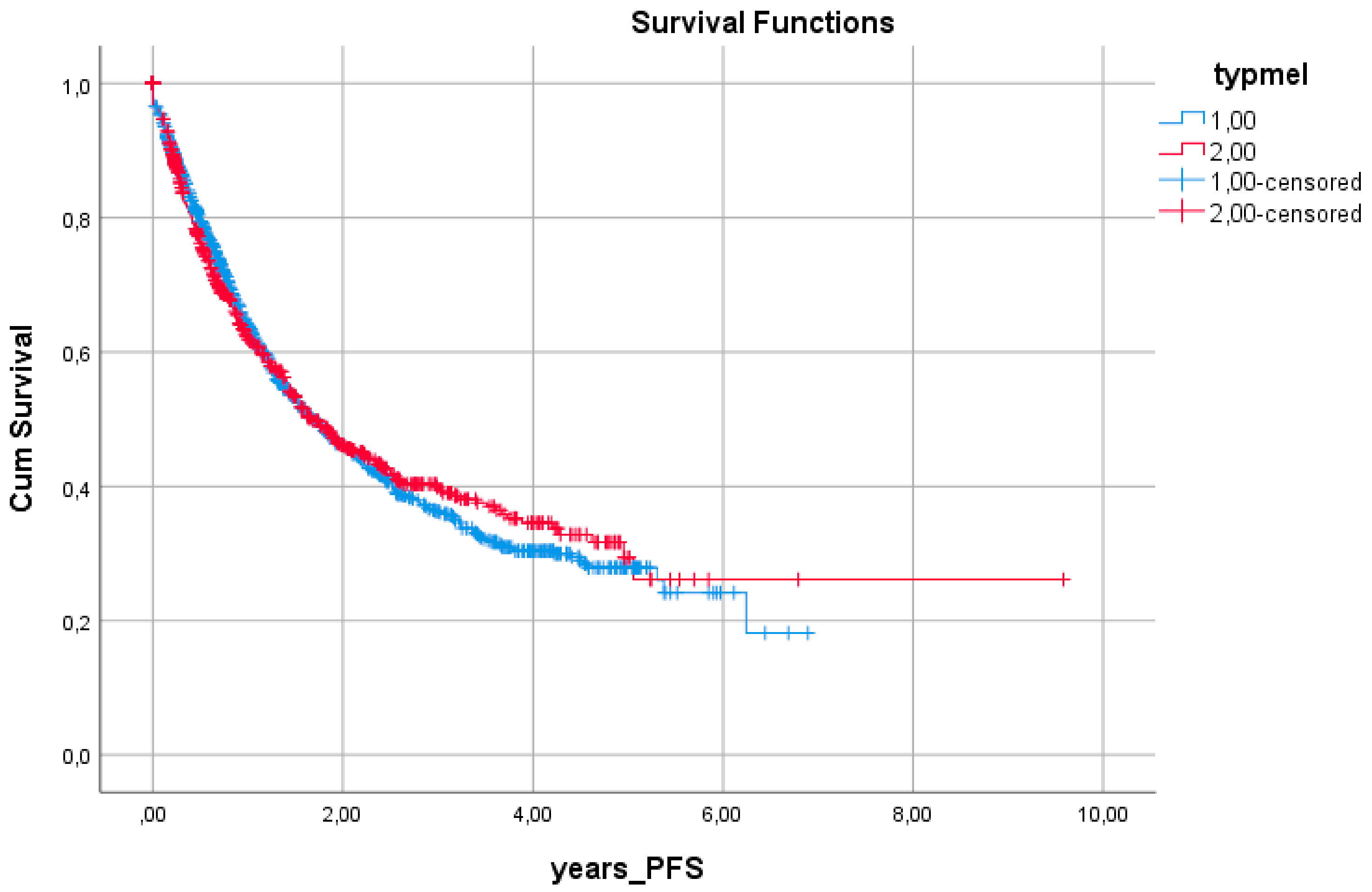

Figure S2. Kaplan Meier curve progression free survival analysis demonstrating the cumulative survival of NM (red) versus SSM (blue) patients treated with immune checkpoint inhibition.

Table S3. Multi variable treatment related cox regression progression free survival analysis in patients treated with immune checkpoint inhibition.

Variables		N	Hazard ratio - 95% CI	P-value
Age		2104	1.00 (0.99 – 1.00)	0.91
Gender	Male	1333	Reference	
	Female	771	0.83 (0.70 – 0.97)	**0.02**
WHO	0 - 1	1902	Reference	
	2 - 4	79	1.02 (0.98 – 1.05)	0.32
Treatment type	Anti-CTLA-4	462	Reference	
	Ant-PD-1 / Anti-CLTA-4	1642	0.91 (0.81 – 1.02)	0.10
LDH	Not elevated	1609	Reference	
	Elevated	458	1.22 (1.13 – 1.32)	**<0.01**
Cerebral disease	Absent	1713	Reference	
	Present	351	1.05 (1.01 - 1.11)	**0.03**
Total organ sites	<3	678	Reference	
	>2	1137	1.01 (0.87 - 1.20)	0.82
Melanoma	SSM	1357	Reference	
	NM	747	0.87 (0.74 – 1.02)	0.09
BRAF mutation	Absent	1053	Reference	
	Present	916	0.85 (0.74 – 0.97)	**0.02**
NRAS mutation	Absent	1112	Reference	
	Present	574	1.12 (1.01 – 1.24)	**0.03**

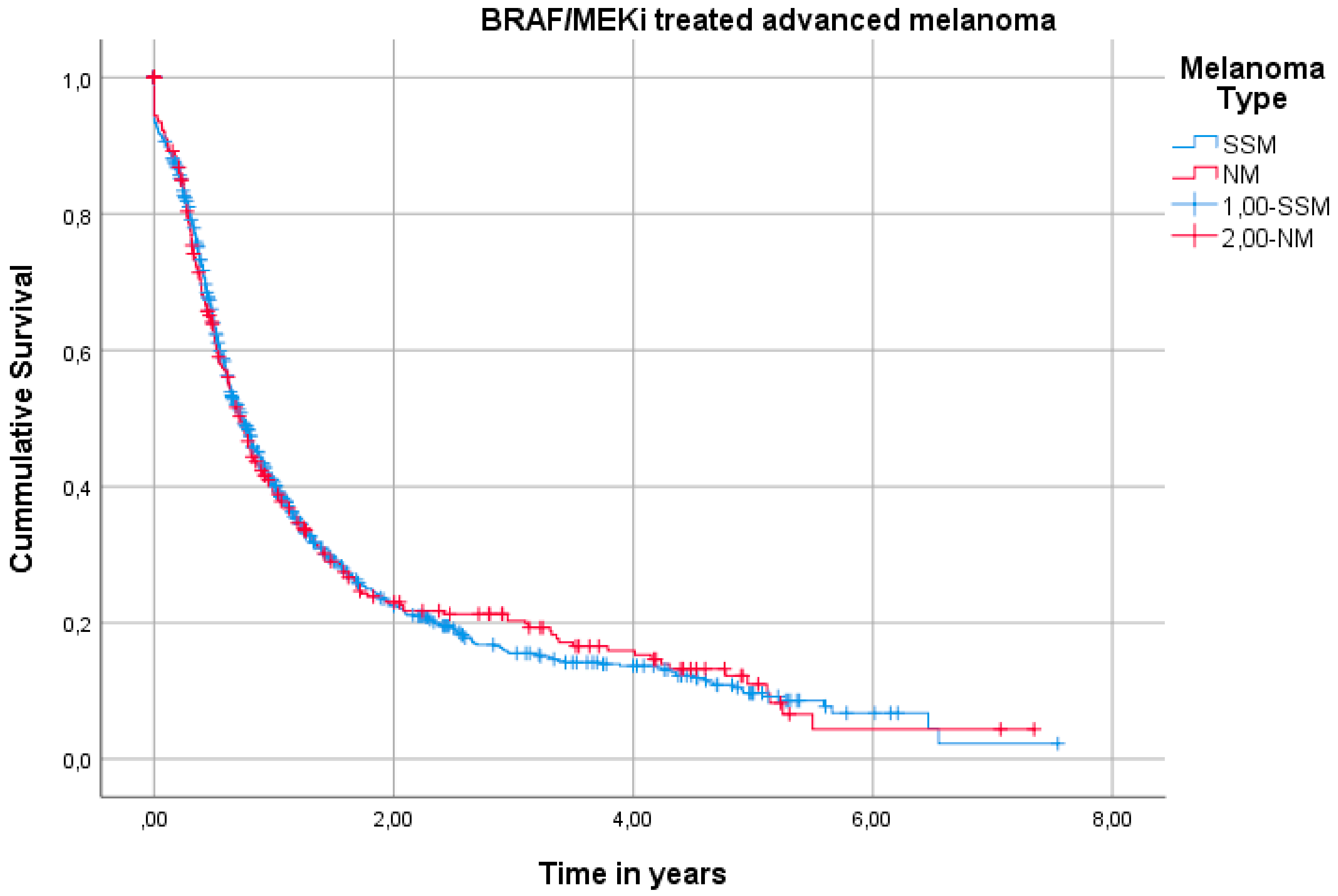

Figure S3. Kaplan Meier curve survival analysis demonstrating the cumulative survival of NM (red) versus SSM (blue) patients treated with immune checkpoint inhibition.

.
